# Palmitoylation as a Functional Regulator of Neurotransmitter Receptors

**DOI:** 10.1155/2018/5701348

**Published:** 2018-04-03

**Authors:** Vladimir S. Naumenko, Evgeni Ponimaskin

**Affiliations:** ^1^Federal Research Center Institute of Cytology and Genetics, Siberian Division of the Russian Academy of Science, Novosibirsk, Russia; ^2^Cellular Neurophysiology, Hannover Medical School, Hannover, Germany

## Abstract

The majority of neuronal proteins involved in cellular signaling undergo different posttranslational modifications significantly affecting their functions. One of these modifications is a covalent attachment of a 16-C palmitic acid to one or more cysteine residues (S-palmitoylation) within the target protein. Palmitoylation is a reversible modification, and repeated cycles of palmitoylation/depalmitoylation might be critically involved in the regulation of multiple signaling processes. Palmitoylation also represents a common posttranslational modification of the neurotransmitter receptors, including G protein-coupled receptors (GPCRs) and ligand-gated ion channels (LICs). From the functional point of view, palmitoylation affects a wide span of neurotransmitter receptors activities including their trafficking, sorting, stability, residence lifetime at the cell surface, endocytosis, recycling, and synaptic clustering. This review summarizes the current knowledge on the palmitoylation of neurotransmitter receptors and its role in the regulation of receptors functions as well as in the control of different kinds of physiological and pathological behavior.

## 1. Introduction

Multiple neurotransmitters of the central nervous system (CNS) act through the activation of a huge variety of different receptors expressed on neurons and glial cells to modulate various aspects of human and animal behavior. The majority of the neurotransmitter receptors can be divided into two groups: (i) metabotropic or G protein-coupled receptors (GPCRs) and (ii) ionotropic receptors or ligand-gated ion channels (LICs) [1]. Signaling properties of the neurotransmitter receptors are under tight control of multiple factors regulating their functional activity and, hence, affecting behavior. One of these factors attracting increasing attention during the last decades includes posttranslational receptor modifications. Prominent examples of such modifications are glycosylation and phosphorylation. In addition, proteins can be modified by the covalent attachment of different lipid moieties such as GPI, myristate, palmitate, and stearate (i.e., protein lipidation). Among different classes of receptor lipidation, a special attention is paid to S-acylation—the covalent attachment of the long-chain fatty acid palmitate or stearate to cysteine residue(s) via thioester linkages. Since the modification with the palmitic acid (palmitoylation) is highly predominant among the S-acylated proteins, we will refer to this modification as palmitoylation throughout the text. In contrast to other types of lipidation, palmitoylation is a dynamic modification, and repeated cycles of palmitoylation/depalmitoylation are known to modulate different protein functions [2]. Generally, more than 70% of all known GPCRs contain the potential palmitoylation site(s) downstream of their seventh transmembrane domain, strongly suggesting that palmitoylation can represent a general feature of neurotransmitter receptors [3]. There are also a lot of experimental data providing direct experimental evidence for palmitoylation of neurotransmitter receptors. In many cases, the functional role of receptor palmitoylation was verified by creation and analysis of palmitoylation-deficient mutants (Table 1). More recently, proteomic approaches applied to study global palmitoylation of neuronal proteins have confirmed palmitoylation of endogenously expressed neurotransmitter receptor under *in vivo* conditions [4, 5]. Disruption of palmitoylation could significantly affect a variety of neurotransmitter receptors properties, including conformation [6, 7], trafficking and localization on the plasma membrane [8, 9], and downstream signaling [10, 11]. This review summarizes our current knowledge on the palmitoylation of neurotransmitter receptors and its role in the regulation of receptors functions and, as consequence, in the control of different kinds of physiological and pathological behavior.

## 2. Enzymology of Receptor Palmitoylation

Palmitoylation is catalyzed by a family of palmitoyl acyltransferases (PATs) that contain a conserved DHHC (Asp-His-His-Cys) cysteine-rich domain directly involved in the palmitoyl-transfer reaction. The DHHC-motif is embedded within a 51-amino acid domain that is a variant of the C2H2 zinc finger motif. A multitude of DHHC proteins exist in eukaryotic cells, seven in yeast and 23 in humans [12–14]. Besides the cysteine-rich domain, little sequence conservation exists between DHHC proteins. Their size varies from 263 to 765 amino acids, and the number of (predicted) transmembrane regions—from four to six [15, 16]. Most DHHC proteins are expressed in multiple tissues, but some of them are expressed in only defined cell types. For example, DHHC2 is expressed only in kidney and testis, whereas DHHC11 is expressed exclusively in testis [17]. The majority of DHHC proteins are localized at endoplasmic reticulum (ER) and Golgi membranes, with a small number targeted to the plasma membrane, endosomes, and synaptic vesicles [18, 19]. Various studies including our own analyzed the alteration in the palmitoylation level of substrate proteins upon overexpression with DHHC proteins or upon shRNA-mediated inhibition of DHHC expression. By the use of such assays, it was shown that most proteins can be palmitoylated by several but not each of the various DHHC proteins, indicating that the 23 enzymes show distinct but overlapping substrate specificities [12, 20–22]. It is assumed that there are multiple recognition elements in a substrate protein and that sequence variation within and outside the cysteine-rich domain determines the substrate specificity of DHHC proteins [23]. In several cases, domains affecting the recruitment of specific substrates were identified experimentally [18, 24–27]. Very recently, two novel noncanonical endocytic signals were identified within the C-terminus of zDHHC2, the PAT responsible for palmitoylation of AKAP79/150 and PSD95 [28]. Mutation of these signals enhanced plasma membrane accumulation of zDHHC2 in both PC12 cells and neurites of rat hippocampal neurons. In addition, authors demonstrated a potential role of phosphorylation for functional modulation of these regulatory domains. Also the first high-resolution crystal structure of the complex between the ankyrin-repeat domain of neuronal DHHC17 and its canonical substrate Snap25b was experimentally solved [29]. This study not only revealed the structural basis of interaction between PAT and its substrate but also demonstrated the role of critical residues for substrate binding and palmitate transfer and show the involvement of the same residues in binding huntingtin, another important substrate of DHHC17 [29]. DHHCs may also differ in their acyl-CoA specificity, explaining S-acylation of proteins with different fatty acids [23, 30]. For example, it was shown that DHHC2 can efficiently transfer acyl-chains of 14 carbon atoms and longer, whereas DHHC3 activity was greatly reduced when acyl-CoAs with chain lengths longer than 16 carbon atoms were provided as substrates. As mentioned above, S-acylated proteins are predominantly modified with palmitate, although protein modifications with stearate, arachidonate, and eicosapentaenoate has been demonstrated as well [31, 32]. To date, PATs responsible for palmitoylation of 6 different neurotransmitter receptors, including 4 GPCRs and 2 LICs, were identified experimentally (Table 1, see also text below).

Palmitoylation can be reversed by the action of thioesterases that remove the acyl moiety bound from the cysteine. Three cytosolic and 2 lysosomal thioesterases have been identified, including the acyl-protein thioesterases APT1, APT2, and APT1-like and the palmitoyl-protein thioesterases PPT1 and PPT2, respectively [33]. Furthermore, recent studies described the isolation of 38 serine hydrolases from mouse (so-called ABHD proteins) with depalmitoylating activity [34, 35]. The physiological importance of the PAT enzymes has been mainly studied in the field of neurobiology, as alterations of their function often result in severe disease, such as Alzheimer's and Huntington's disease, schizophrenia, and mental retardation [18]. Thus, DHHCs represent a potential target for treatment of multiple diseases, and drugs against DHHC proteins are currently under development [36]. At the same time, there are some data on the functional roles of palmitoylation of different neurotransmitter receptors, indicating the involvement of this modification in the mechanisms underlying different types of normal and pathological behavior. For example, it was shown that palmitoylation of the NMDARs could play a role in the mechanisms of pain [37], and palmitoylation of defined AMPA receptor subunits controls psychomotor sensitivity to the psychoactive drug *in vivo* via regulation of receptor trafficking and subcellular localization [9].

## 3. Palmitoylation of G Protein-Coupled Receptors (GPCRs)

### 3.1. Adrenergic Receptors

Adrenoreceptors belong to the GPCR adrenoceptor family and are activated by the catecholamines norepinephrine and epinephrine. Based on their pharmacological characteristics, adrenoceptors were originally divided into two major types, alpha and beta. The current classification includes three major receptor types, alpha-1 adrenoceptors (*α*1ARs), alpha-2 adrenoceptors (*α*2ARs), and beta adrenoceptors (*β*ARs) [38]. The *α*1ARs are coupled to Gq protein, while *α*2ARs receptors activate inhibitory Gi protein. The *β*ARs, which are composed of three subtypes *β*1, *β*2, and *β*3, are linked to Gs proteins, although the *β*2AR isoform also couples to inhibitory Gi proteins. Adrenoreceptors are implicated in diverse physiological functions, including modulation of the cardiovascular, endocrine, renal, and pulmonary systems [39]. In the CNS, adrenoreceptors are involved in the regulation of different kinds of normal and pathological behavior, including aggression [40], sexual behavior [41], emotional regulation of pain [42], attention-deficit/hyperactivity disorder [43], drug addiction [44], antidepressant drug action [45], and Parkinson's disease [46].

The *β*2AR was the first GPCR for which palmitoylation was demonstrated experimentally [47]. After this pioneering study, multiple aspects of *β*2AR palmitoylation were investigated in great details in more than 20 follow-up publications. In the initial work by O'Dowd et al., authors identified Cys341 in the receptor's C-terminal domain as a palmitoylation site and demonstrated a crucial role of *β*2AR palmitoylation in the normal coupling of the receptor to the adenylyl cyclase (AC) signaling. A follow-up study by the same group demonstrated that receptor stimulation leads to transient increase in palmitoylation mediated by the increased palmitate turnover [10, 11]. It is noteworthy that palmitoylation of the *β*2AR at Cys341 as well as agonist-mediated increase in palmitate turnover was recently confirmed by the mass spectrometric analysis [48]. Moreover, nonpalmitoylated *β*2AR mutant has undergone an increased phosphorylation and is uncoupled from Gs protein. Also the receptor desensitization was largely affected by the disruption of palmitoylation [49–51]. In addition, palmitoylation of *β*2AR seems to play an important role in complex building between receptor and *β*-arrestin [52]. Palmitoylation of *β*2AR is also involved in the modulation of *β*-adrenergic signaling pathway by nitric oxide (NO) in the way that NO decreased receptor palmitoylation [53]. Surprisingly that after more than 25 years' intensive analysis of *β*2AR palmitoylation, Adachi et al. recently identified Cys265 within the third intracellular loop as a novel palmitoylation site [8]. While basal palmitoylation of Cys265 is extremely low, receptor stimulation results in enhanced palmitoylation at this position. Functionally, palmitoylation of Cys265 may stabilize receptor at the plasma membrane, thus playing a role in the *β*2AR trafficking and localization. Palmitoylation of this atypical cysteine residues is mediated by the Golgi-resident palmitoyl transferases zDHHC9/14/18 and is followed by depalmitoylation via the plasma membrane-localized acyl-protein thioesterase APT1 [8]. Because Cys265 is not conserved in *β*1AR, the authors suggested that selective palmitoylation of *β*2AR at Cys265 may be associated with functional differences between *β*2AR and *β*1AR, in particular with those dealing with resistance of *β*2AR to downregulation.

The palmitoylation of the adrenergic receptors belonging to the *α*2AR group was extensively investigated as well. In 1993, Kennedy and Limbird demonstrated that *α*2AR is palmitoylated and that replacement of C-terminal Cys442 eliminates detectable palmitoylation without perturbing receptor coupling to Gi protein [54]. In the follow-up study, the same authors demonstrated that palmitoylation of the *α*2AR is a dynamic process regulated by agonist, and that sequence distal to Cys442 is not required for palmitoylation [55]. Surprisingly, regulation of receptor palmitoylation by agonist was not confirmed in the most recent study, in which palmitoylation of the fusion protein between the *α*2AR and the alpha subunit of the Go protein was analyzed [56]. In this study, the authors found that regulation of palmitoylation by the agonist occurred only for the G protein. Moreover, functional analysis of the *α*2AR palmitoylation showed that palmitoylation was specifically involved in agonist-promoted receptor downregulation upon chronic agonist exposure without affecting other receptor functions [57].

More recently, *α*1AR has also been shown to undergo palmitoylation [58]. In this study, authors demonstrated that zDHHC21 forms a complex with the *α*1DAR and can thus be PAT responsible for the receptor palmitoylation. Analysis of the vascular functions in a mouse expressing a nonfunctional ZDHHC21 (F233Δ) revealed diminished functions of vascular *α*1AR, leading to hypotension and tachycardia mediated by the reduced vascular tone [58]. Although this study was focused on the functional role of palmitoylation of peripheral *α*1ARs, it can also have implications for *α*1ARs expressed in the brain, where these receptor are critically involved in the regulation of cognitive functions and behavioral activation [59].

### 3.2. Serotonin Receptors

Serotonin (5-hydroxytryptamine or 5-HT) is an important neurotransmitter involved in the regulation of different physiological functions, including most of the forms of normal and pathological behavior. Multiple effects of 5-HT are mediated by the existence of 14 serotonin receptor subtypes. With exception of the 5-HT_3_ receptor, that is, LIC, all other 5-HT receptors belong to the GPCR family. Palmitoylation of four 5-HT receptors, including 5-HT_1A_, 5-HT_1B_, 5-HT_4_, and 5-HT_7_, was experimentally confirmed. More recently, proteomic approaches using a global, site-specific analysis of neuronal protein S-acylation have uncovered 5-HT_2A_ receptor as a putative palmitoylated protein [5]. Moreover, 5-HT_2B_ and 5-HT_6_ receptors contain potential palmitoylation sites within their C-terminus, although palmitoylation of these receptors was not demonstrated experimentally.

Among serotonin receptors, the 5-HT_1A_ receptor attracts particular attention because of its key role in the regulation of the brain 5-HT system functioning [60]. There are also a lot of data on the role of 5-HT_1A_ receptor in the mechanisms underlying aggression [61], anxiety [62], depression [62, 63], depressive psychosis [64], and suicidal behavior [65]. The 5-HT_1A_ receptor is palmitoylated at its C-terminal Cys417 and Cys420 residues [66]. In contrast to other palmitoylated GPCRs that usually undergo repeated cycles of palmitoylation/depalmitoylation, 5-HT_1A_ receptor palmitoylation is irreversible and insensitive to the agonist stimulation [66]. Functionally, palmitoylation of 5-HT_1A_ receptor is necessary for its communication with the G*α*i-subunits. Furthermore, nonpalmitoylated mutants failed to inhibit cAMP formation and activate mitogen-activated protein kinase (MAPK), demonstrating the importance of receptor palmitoylation for the downstream effector signaling [66]. The underlying molecular mechanism might include mislocalization of the receptor within the plasma membrane: While the wild-type 5-HT_1A_ receptors are preferentially localized within the membrane rafts, nonpalmitoylated mutants are excluded from this membrane subdomain [67], which is known to act as a signaling “hot spot” [68].

In addition to the 5-HT_1A_ receptor, the 5-HT_1B_ receptor has also been shown to be palmitoylated [69]. Although the functional role of 5-HT_1B_ receptor palmitoylation was not experimentally elucidated, the high homology between 5-HT_1A_ and 5-HT_1B_ receptors suggests that palmitoylation of the 5-HT_1B_ receptor can be critically involved in the regulation of its functions.

Another palmitoylated serotonin receptor is the 5-HT_4_ receptor. In the mammalian brain, the 5-HT_4_ receptor is involved in the control of acetylcholine and dopamine secretion, facilitates cognitive performance, and is also implicated in learning and memory. This receptor also plays a role in the mechanisms underlying anxiety [70], neurodegenerative diseases, major depressive disorder, and anorexia [71, 72]. Palmitoylation of the 5-HT_4_ receptor is a reversible modification, and receptor stimulation increases the turnover rate for receptor-bound palmitate [73]. In addition to highly conserved cysteine residue Cys328/Cys329, presented in all 5-HT_4_ receptor isoforms, Cys386 within the C-terminal domain of 5-HT_4(a)_ receptor isoform was identified as an additional palmitoylation site [74]. From the functional point of view, 5-HT_4_ receptor palmitoylation has been shown to not be involved in the G protein coupling as well as in receptor trafficking. However, mutation of Cys328/Cys329 resulted in a significant increase of the receptor's constitutive activity, suggesting that dynamic palmitoylation of the 5-HT_4_ receptor could affect isomerization of the receptor from inactive to active form by formation of an additional cytoplasmic loop [68]. It is noteworthy that the C328S/C329S mutant exhibited enhanced receptor phosphorylation under basal conditions and after agonist stimulation. This mutant was also more effectively desensitized and internalized via a *β*-arrestin-mediated pathway, when compared with the wild-type receptor [68].

More recently, we have found that the mouse 5-HT_7(a)_ receptor isoform undergoes palmitoylation as well [75]. The 5-HT_7_ receptor is associated with a number of physiological and pathophysiological responses, including age-dependent changes of the circadian timing [76] and phase shifting of the circadian rhythm [77]. In addition, a large body of evidence indicates an involvement of the 5-HT_7_ receptor in the development of anxiety and depression [78]. At the cellular level, the 5-HT_7_ receptor was shown to modulate the neurite outgrowth, synaptogenesis, and neuronal excitability [79, 80]. The 5-HT_7_ receptor is dynamically palmitoylated in an agonist-dependent manner, and its C-terminal cysteine residues Cys404 and Cys438/Cys441 were identified as potential palmitoylation sites [75]. Functional analysis of palmitoylation-deficient mutants revealed that palmitoylation of the 5-HT_7_ receptor was not involved in an agonist-induced activation of Gs and G12 proteins. In contrast, mutation of the Cys404 (either alone or in combination with Cys438 and Cys441) resulted in significantly increased constitutive, agonist-independent 5-HT_7_ receptor activity. It is noteworthy that only the activation of Gs-mediated pathway was increased, whereas the activation of G*α*12-protein was not affected [75].

### 3.3. Dopamine Receptors

Dopamine plays a crucial role in the regulation of various physiological processes, including executive function, learning, reward, motivation, and neuroendocrine control [81]. Dysfunction of dopaminergic signaling may be involved in multiple neuronal disorders such as central fatigue [82], tardive dyskinesia [83], Parkinson's [84] and Alzheimer's disease [85], major depression [86], and schizophrenia [87]. Moreover, medications which increase the dopamine level in the brain by inhibiting dopamine reuptake or stimulation of defined dopamine receptors have been proven to be potent antidepressants [88, 89].

Dopamine operates through the activation of five distinct GPCRs that are divided into two major groups: D1 and D2. The D1 family consists of D1 and D5 receptors, which are coupled to the stimulatory Gs protein to activate AC. The members of the D2 family, including D2, D3, and D4 receptors, mediate Gi protein-mediated inhibition of AC [90]. Palmitoylation of the recombinant D1 receptor was demonstrated more than 20 years ago using an overexpression of recombinant receptor in the baculovirus system [91]. In the same study, the authors have shown that receptor stimulation resulted in an increased level of [^3^H]-palmitate incorporation into the receptor. Studies on the functional role of D1 receptor palmitoylation revealed that substitution of palmitoylated cysteines Cys347 and Cys351 by alanine failed to affect the receptor affinity for agonists as well as receptor ability to stimulate AC [92, 93]. More recently, Kong et al. demonstrated that palmitoylation of D1 receptor is critically involved in agonist-dependent receptor internalization [94].

Both short and long isoforms of D2 receptor (D2S and D2L) have also been shown to be palmitoylated [95, 96]. Functionally, palmitoylation of D2L, which takes place at Cys443, is involved in the regulation of receptor stability and trafficking to the plasma membrane [97]. In this study, palmitoyl acyltransferase (PAT) DHHC4 was identified as a D2L interaction partner, suggesting that this PAT might be responsible for receptor palmitoylation. The D3 receptor also undergoes posttranslational palmitoylation, which is involved in the regulation of multiple receptor functions, including cell surface expression, protein kinase C-mediated endocytosis, agonist affinity, and agonist-induced receptor tolerance [98]. This study also revealed that although C-terminal domains of D2 and D3 receptors possess a high homology, D3 receptor is palmitoylated more extensively. Based on these findings, authors suggested that regulated palmitoylation may represent a new strategy for selective modulation of D3 receptor. This assumption is extremely important due to the fact that D2 and D3 receptors are the main targets of currently used neuroleptic drugs. The most serious side effects of the currently used antipsychotics are disturbances in motor functions [99]. Since D2 and D3 receptors are heavily expressed in the regions responsible for motor and emotion-related mental functions, respectively, development of D3 receptor-specific ligands or selective manipulation of the specific signaling pathways of D3 receptor can be used as a strategy to separate the desired therapeutic antipsychotic activities from side effects on motor function.

Also the last member of the D2 receptor family, D4 receptor, is palmitoylated on its terminal cysteine residue Cys467. Similar to that of the D3 receptor, palmitoylation of D4 receptor has been shown to regulate a receptor's cell surface expression, signaling, and endocytosis [100].

### 3.4. Vasopressin Receptors

The hormone arginine vasopressin (AVP), which is produced in the neurohypophysis, plays an important role in a wide range of physiological functions, including water reabsorption, cardiovascular homeostasis, and endocrine functions [101]. AVP works not only as a peripheral hormone but also as a neuropeptide influencing multiple brain functions such as regulation of memory [102]; maternal care and anxiety-related behavior [103]; and parental, social, and sexual behavior [104, 105] as well as aggressive behavior [106]. These and other actions of AVP are mediated by three vasopressin receptor subtypes—V1a, V1b, and V2—whereas only V1 receptor isoforms are expressed in the CNS (with the exception of an autoregulatory V2 receptor expressed exclusively on the AVP neurons) [107]. V1a and V1b receptors couple to the Gq protein to regulate phospholipase C (PLC) pathway, and the V2 receptor couples to the Gs protein [101].

The V1a receptor (V1aR) is palmitoylated on Cys371 and Cys372 located in the C-terminal receptor domain [108], and palmitoylation of the V1aR is a reversible modification regulated by AVP. While receptor affinity to ligands as well as intracellular signaling was not affected by the nonpalmitoylated mutants, receptor phosphorylation under both basal and AVP-stimulated conditions was abolished compared to the wild-type V1aR. Moreover, the nonpalmitoylated receptor was sequestered at a faster rate [108].

In the vasopressin V2 receptor (V2R), conserved cysteine residues at positions 341 and 342 were identified as putative acylation sites [109, 110]. The V2R is palmitoylated on both cysteine residues, and each cysteine is palmitoylated independently from the other [109, 110]. Functional analysis of palmitoylation-deficient mutants revealed that ligand binding affinity, AVP-induced AC stimulation, receptor internalization, and desensitization were not affected by the absence of palmitoylation [109, 110]. However, V2R palmitoylation was important for the intracellular receptor trafficking and localization on the plasma membrane [109]. A later study by Charest and Bouvier demonstrated that prevention of the V2R palmitoylation by site-directed mutagenesis significantly slows down agonist-promoted receptor endocytosis. Moreover, V2R-mediated activation of MAPK was reduced in the case of nonpalmitoylated receptor mutant [111]. The authors also investigated a possible interplay between receptor endocytosis and vasopressin-stimulated MAPK activity and found that the reduced kinase activation obtained by the palmitoylation-deficient mutant was not mediated by the altered receptor endocytosis [111].

### 3.5. Adenosine Receptors

Adenosine acts as a neurotransmitter in the brain by activating four specific GPCRs, including A1, A2A, A2B, and A3 receptors. Stimulation of the A2A and A2B adenosine receptors (ARs) increases cAMP production via Gs protein, resulting in activation of protein kinase A (PKA) and phosphorylation of the cAMP response element binding protein (CREB). In contrast, activation of the A1 and A3 ARs inhibits cAMP production and decreases PKA activity and CREB phosphorylation [112]. In addition, A1AR can facilitate phospholipase C (PLC) activity through the activation of the Gq protein. ARs are known to be involved in the mechanisms underlying different forms of normal and pathological behavior, including social behavior and anxiety [113, 114], avoidance learning [115], mood and memory dysfunction triggered by chronic stress [116], and cognitive impairment [117].

The human A1AR is palmitoylated at the C-terminal cysteine residue Cys309, and the palmitoylation level is not influenced by the agonist stimulation [118]. It is noteworthy that various receptor functions, including the kinetics of agonist-induced receptor downregulation, coupling of receptor to Gi protein, and activation of the downstream effectors, such as GIRK1 and CIR K^+^ channels, were not affected by substitution of this cysteine with either serine or alanine [118]. Interestingly, substitution of the homologous cysteine residue Cys305 within the C-terminus of the A3AR resulted in significantly faster agonist-mediated internalization of this mutant compared with the wild type, although analogous mutation of the human A1AR (Cys309Ala) had no effect on receptor internalization [119]. Moreover, unlike the wild-type A3AR and nonpalmitoylated A1AR, the entire pool of internalized nonpalmitoylated A3AR mutant was able to recycle back to the plasma membrane after agonist removal. This effect was mediated by the subtype-specific differences in the *β*-arrestin recruitment correlating with the sensitivity of the receptor's C-terminus to GRK phosphorylation. Together, these results suggest that in the case of the A3AR, sensitivity to GRK-mediated internalization might be regulated by the palmitoylation upstream of the GRK phosphorylation [119, 120].

The A2BAR also contains conserved cysteine residue at position 311, which has been suggested to undergo palmitoylation. Homology modeling, molecular docking, and molecular dynamic simulations of A2BAR revealed that palmitoylation can bend the proximal portion of the receptor's C-terminal tail toward the membrane, leading to a deeper insertion into the lipid bilayer [121]. In addition, palmitoylation of A2BAR can modulate the structure of the last transmembrane domain, which is essential for interaction with different proteins related to sorting and signaling of A2BAR.

### 3.6. Opioid Receptors

Opioid system plays a pivotal role in the modulation of pain behavior and antinociception. Opioid receptors are expressed in multiple CNS regions, including reward and emotion-related brain structures [122]. In addition to their well-known antinociceptive effects, opioid receptors are known to play a role in the mechanisms of drug abuse [123] and alcohol addiction [124]. There is also some evidence on involvement of opioid receptors in the mechanisms of antidepressant drug action [125]. Currently, four different opioid receptor isoforms, including mu (*μ*), delta (*δ*), kappa (*κ*), and opioid receptor like-1 (ORL1), have been extensively characterized at the cellular, molecular, and pharmacological levels. All four opioid receptor types couple to pertussis toxin-sensitive Gi protein to cause inhibition of cAMP formation [122].

The rat *μ*-opioid receptor (MOR) has been shown to be palmitoylated, and receptor activation with morphine did not modulate the extent of receptor palmitoylation [126]. Surprisingly, mutations of the two conserved cysteine residues (i.e., Cys346 and Cys351) in the receptor's C-terminus do not affect [^3^H]palmitic acid incorporation, suggesting that, unlike that of the other GPCRs, palmitoylation of the MOR does not take place in its C-terminal domain. More recently, cysteine residue at position 170 was identified as a palmitoylation site of the MOR [127]. Using both experimental analysis and computational models, the authors demonstrated that receptor-bound palmitate can interact with the membrane cholesterol to facilitate receptor homodimerization and G protein coupling/activation. When cholesterol metabolism or receptor palmitoylation was affected, stability of homodimers became altered, leading to the uncoupling of G protein. Therefore, the authors suggested that the cellular cholesterol content can represent an additional target for regulation of the receptor-mediated signaling [127]. In a more recent study, palmitoylation of the MOR was confirmed using a nonisotopic bioorthogonal click chemistry [128]. Moreover, the authors demonstrated that two PATs, zDHHC3 and zDHHC4, are capable of interacting with and palmitoylating the MOR.

The other member of opioid receptor family, the *δ*-opioid receptor (DOR), undergoes posttranslational palmitoylation at two different cellular locations [129]. The newly synthesized DOR is constitutively palmitoylated during its transport to the cell surface, and this initial palmitoylation is needed for the efficient trafficking. After reaching the cell surface, the receptor-bound palmitate turns over rapidly in an agonist-dependent manner. It is noteworthy that the agonist-mediated turnover of DOR palmitoylation did not affect receptor-G protein coupling and internalization/recycling of the receptor [129].

### 3.7. Cannabinoid Receptors

The endocannabinoid system consists of endogenous cannabinoids, synthetic/degradative enzymes regulating the levels of endocannabinoids, and at least two GPCRs known as the cannabinoid type 1 and type 2 receptors (CB1R and CB2R) [130]. Both CB1 and CB2 receptors couple to the G proteins of Gi/o family. However, under certain conditions, coupling of this receptors to Gs and Gq/11 proteins was also demonstrated [131]. Endocannabinoid signaling critically regulates emotional and motivational states via activation of CB1R in the brain [132]. It is also involved in responses to stress [133], sleep-wake cycle, and mood [134]. Moreover, there are some evidence that the endocannabinoid system could modulate the functional activity of other brain neurotransmitter systems. For example, activation of CB1 receptor (CB1R) leads to activation of dopaminergic neurons [135].

It has been shown that endogenous CB1R in the rat brain undergoes palmitoylation and that mutation of C-terminal Cys415 residue prevents receptor palmitoylation [136]. It is noteworthy that recruitment of the CB1R to the plasma membrane as well as receptor localization at lipid rafts was impaired in nonpalmitoylated mutants, indicating the role of palmitoylation in receptor trafficking and localization. In addition, nonpalmitoylated CB1R significantly reduced receptor association with G*α*i-subunit, demonstrating that palmitoylation may be responsible for the functional transmission of the agonist-induced conformational changes from receptors to the G protein [7]. Similar to that of the MOR, palmitoylation of the CB1R seems to be involved in the modulation of the conformational state of helix 8 and interactions of the CB1R with cholesterol and caveolin 1 [6]. These combined results suggest that via interaction with cholesterol, palmitoylation of the CB1R may tune receptor interaction with G proteins and thus act as a targeting signal for its functional regulation.

### 3.8. Muscarinic Acetylcholine Receptors

Muscarinic acetylcholine (ACh) receptors belong to the GPCR family and consist of five distinct receptor subtypes (M1 to M5). All five muscarinic receptor subtypes are expressed in the brain, where they activate a multitude of signaling pathways modulating synaptic plasticity, neuronal excitability, and feedback regulation of ACh release [137]. Central muscarinic receptors are also involved in higher cognitive processes such as learning and memory [138]. The brain M2 ACh receptor is known to contribute to impaired cognitive function, including a deficit in behavioral flexibility, working memory, and hippocampal plasticity [139, 140]. M1 receptors are most abundant in the neocortex, hippocampus, and neostriatum; M4 receptors are highly expressed in the neostriatum; and M5 receptors are localized on the projection neurons of substantia nigra, pars compacta, and the hippocampus. While M2 receptors are distributed throughout the whole brain, levels of M3 receptors are relatively low [138].

All five muscarinic ACh receptor subtypes contain a conserved cysteine residue within the receptor's C-terminus in the homologous position. Mutation of the Cys457 in M2 receptor was shown to have a little effect on receptor-mediated AC inhibition [141]. Similarly, mutation of the C-terminal cysteine residue in M1 receptor did not affect the ligand binding or agonist-mediated phosphatidylinositol turnover [142]. Unfortunately, the authors of the above-mentioned studies did not provide experimental evidence for palmitoylation of M1 and M2 receptors. Such analysis was carried out by Hayashi and Haga, who reported that M2 receptor is metabolically palmitoylated at Cys457 [143]. For the functional analysis, nonpalmitoylated receptor mutants were purified and reconstituted together with G proteins into phospholipid vesicles. These experiments revealed that disruption of M2 receptor palmitoylation failed to affect receptor interaction with Gi/o proteins, whereas Gi_2_-mediated signaling was reduced in mutant receptors. These results suggest that although the M2 receptor palmitoylation is not required for G protein coupling, it can enhance the ability of the receptors to interact with G proteins.

### 3.9. Neuropeptide Y Receptors

Although the functional role of neuropeptide Y (NPY) is investigated to significantly lower extent as that for the brain serotonin, dopamine or noradrenaline, it is known that this neuropeptide could regulate levels of monoamines and corticosterone [144]. Neuropeptide Y acts through the specific NPY receptors (Y_1_, Y_2_, Y_4_, and Y_5_), which mediate various physiological functions and are involved in several human diseases, such as obesity, hypertension, epilepsy, metabolic disorders, and cancer [145]. Moreover, there are some data on the role of neuropeptide Y in the mechanisms of depression [146, 147], suggesting a possible cross-talk between this neuropeptide and the brain 5-HT system. Activation of NPY receptors leads to the inhibition of AC as well as to modulation of Ca^2+^ and K^+^ channels [148].

Until now, palmitoylation was experimentally confirmed only for the Y_1_ receptor [149]. By site-directed mutagenesis, C-terminal Cys337 was identified as a single palmitoylation site on the Y_1_ receptor. While replacement of this cysteine by alanine or serine did not influence the high-affinity binding sites of agonists and antagonists, acylation-deficient receptor was unable to couple with corresponding G protein. Moreover, palmitoylation influenced receptor desensitization [149]. In general, results of this study suggest that the endogenous regulation of Y_1_ receptor palmitoylation may be a significant mechanism for control of balance between G protein activation and receptor desensitization.

### 3.10. Melatonin Receptors

The hormone melatonin secreted during darkness from pineal gland provides a circadian signal to the organism in vertebrates [150, 151]. Besides its circadian regulatory functions, melatonin is involved in a variety of physiological responses including vasoactive, visual, and neuro-immunological properties, and it also possesses neuroprotective effects [152, 153]. Melatonin is implicated in a variety of diseases, including cancer and Alzheimer's disease [154]. Effects of melatonin are mediated via two specific GPCRs: MT1 and MT2 [155]. In the single study on melatonin receptor palmitoylation, Sethi and coauthors suggested that both M1 and MT2 receptors can be palmitoylated on conserved C-terminal cysteine residues Cys7.72 and Cys7.77, respectively [156]. Mutation of these putative palmitoylation sites blocked receptor-mediated signaling toward cAMP inhibition in each of the melatonin receptor subtypes without affecting agonist binding and agonist-induced receptor internalization [156].

## 4. Palmitoylation of Ligand-Gated Ion Channels (LICs)

### 4.1. Glutamate Receptors

Whereas palmitoylation of GPCRs was systematically investigated starting from the late 1980s, information on palmitoylation of neuronal LICs is much less abundant. One exception is the palmitoylation analysis of ionotropic glutamate receptors (i.e., AMPARs, NMDARs, and kainate), which all are known to undergo palmitoylation.

Glutamate is the major excitatory neurotransmitter in the mammalian CNS, which operates through activation of both metabotropic and ionotropic receptors. The ionotropic glutamate receptors (GluRs) are classified into several groups, including AMPA, NMDA, kainate, and delta receptors [157, 158], with AMPA and NMDA receptors representing more functionally important classes. These receptors play a crucial role in synaptic plasticity, synaptogenesis, and excitotoxicity [159]. They are also involved in a wide range of pathological behaviors, such as drug addiction [160], alcohol abuse [161, 162], anxiety [163], fear-related behavior [164], and epilepsy [165], as well as in mechanisms underlying development of schizophrenia [166]. In addition, these receptors are involved in the regulation of pain sensation [167].

#### 4.1.1. Palmitoylation of AMPA Receptors

AMPA receptors (AMPARs) are mainly responsible for the fast excitatory transmission at central synapses. These receptors are constantly internalized, recycled, and inserted in the synapses, and such dynamic trafficking represents a main molecular mechanism for regulation of synaptic strength and synaptic plasticity [168, 169]. AMPA receptors consist of four subunits (GluA1–GluA4) containing short cytoplasmic tails critical for regulation of their trafficking. All four AMPAR subunits possess two conserved C-terminal cysteine residues. One of them is located in the second transmembrane domain (TMD2) of the receptor, whereas the other one resides in the C-terminal region. Both cysteines can be palmitoylated in cultured neurons as well as receptors endogenously expressed in the forebrain [4, 170]. Palmitoylation of all four AMPAR subunits was also confirmed in the nucleus accumbens (NAc) of adult rats [9]. A later study revealed that cocaine can regulate trafficking of the receptor in NAc neurons and control the response to the psychoactive drug *in vivo* via selective palmitoylation of AMPAR subunits [9]. In combination with an earlier report on glutamate-dependent AMPA receptor depalmitoylation [170], these results suggest that palmitoylation of AMPARs takes place in multiple brain regions and can be dynamically regulated by extracellular signals.

It is noteworthy that palmitoylation of cysteines localized in TMD and in C-terminal receptor domains seems to have different functions. Palmitoylation of cysteine residue within the TMD2 caused receptor trapping in the Golgi apparatus, suggesting that palmitoylation of this cysteine is involved in the quality-control process during the receptor trafficking. A Golgi apparatus-specific protein with a DHHC zinc finger domain (GODZ) was reported to have a PAT activity for the AMPAR [170]. In the same study, GODZ- (DHHC3-) mediated palmitoylation of TMD2 cysteine was reported to disrupt the interaction of receptors with 4.1N, a synapse-enriched cytoskeletal protein that stabilizes surface AMPAR expression and enhances susceptibility to agonist-induced internalization. Depalmitoylation of this cysteine increases the receptor affinity for 4.1N and stabilizes the receptor on surface membrane [170]. Although the role of GODZ in AMPAR palmitoylation was confirmed by the overexpression of this DHHC as well as by introduction of the dominant negative mutation, it is still not clear whether the GODZ represents a main PAT for the endogenously expressed AMPARs. Indeed, a recent study by Fang and coauthors demonstrated that knock-down of GODZ reduces GABA-mediated but not glutamatergic transmission [171].

More recent studies revealed that palmitoylation of GluA1 subunit requires its dynamic anterograde transport from the ER to the Golgi apparatus, while GluA2 subunits are palmitoylated by the ER-resided DHHC2 [172]. Since the majority of palmitoylated GluA2 subunits were not associated with GluA1 subunits, prevention of palmitoylation resulted in a loss of mature GluA2 subunit without affecting GluA1. In addition, pharmacological inhibition of neuronal activity increased the pool of palmitoylated GluA2, without affecting the palmitoylation levels of GluA1 [172].

#### 4.1.2. Palmitoylation of NMDA Receptors

NMDA receptors are heteromeric complexes composed of the obligatory NR1 subunit in combination with NR2A or NR2B, representing the major NR2 subunits in forebrain [173–175]. Unlike the majority of GPCRs, which are palmitoylated on one or two conserved cysteine residues within the C-terminal domain, palmitoylation of NMDARs takes place at two distinct cysteine clusters within the long intracellular C-terminal domain of GluN2A and GluN2B receptor subunits [4, 176]. The first cluster is proximal to the membrane (GluN2A: C848, C853, and C870; GluN2B: C849, C854, and C871). The second cluster resides in the middle of the C-terminus (GluN2A: C1214, C1217, C1236, and C1239; GluN2B: C1215, C1218, C1239, C1242, and C1245). Similar to palmitoylation of AMPARs, NMDAR palmitoylation can be regulated by neuronal activity, although responsible PATs are not yet identified. As with AMPARs, functional consequences of palmitoylation of each cluster within the NMDARs differ markedly. Palmitoylation of the first cysteine cluster on a membrane-proximal region leads to stabilizing the NMDARs at the cell surface [176]. This can be mediated by the palmitoylation-dependent increase in tyrosine phosphorylation of NR2A and NR2B via Fyn-dependent Src protein tyrosine kinase (PTK). At the molecular level, palmitoylation of Cys cluster I may, via DHHC3, modulate conformation of the NR2B C-terminus, facilitating the accessibility and/or interaction of Src-family PTKs to their substrate [176]. In addition, palmitoylation of this cluster regulates constitutive NMDA receptor internalization in developing neurons. Palmitoylation of the second cluster leads to accumulation of both NR2A- and NR2B-containing receptors in the Golgi apparatus, the effect quite similar to that obtained for the AMPARs. More recently, functional synaptic expression of NMDARs containing mutations of cysteine clusters was assessed by Mattison and coworkers [177]. This study demonstrated that mutation of cysteine residues within the membrane-proximal cluster causes the decrease in the synaptic expression of NMDARs mediated by enhanced receptor internalization. In contrast, mutation of the cysteines located in the middle part of the GluN2A and GluN2B C-terminus failed to alter the synaptic pool of NMDA receptors [177]. These combined results point out palmitoylation as an additional fine-tuning regulator of NMDAR trafficking

#### 4.1.3. Palmitoylation of Kainate Receptors

Ionotropic kainite glutamate receptor GluR6 was the first ionotropic receptor shown to be palmitoylated [178]. Two cysteine residues (C827 and C840) in the putative intracellular C-terminal domain of the receptor were identified as palmitoylation sites. Sequence alignment performed in this study revealed that C827 of the GluR6 is conserved in all of the kainate receptors except GluK5, whereas C840 is conserved only in the mammalian kainate receptor subtypes [178]. While the current properties of the double mutant were not changed in comparison to those of the wild-type receptors, nonpalmitoylated receptors undergo stronger PKC-mediated phosphorylation. More recently, palmitoylation of the GluK2 kainate receptor subunit has been shown to promote 4.1N association, whereas PKC phosphorylation antagonizes this interaction [179]. Together with the result obtained for the AMPAR palmitoylation, these data suggest that palmitoylation of AMPA and kainite receptors differentially regulates their insertion and stabilization at the cell surface. More importantly, modulation of their association with 4.1N by palmitoylation and phosphorylation might represent a central mechanism for distinct functions of these receptors in the brain.

### 4.2. Gamma-Aminobutyric Acid (GABAA) Receptors

Gamma-aminobutyric acid (GABA) receptors belong to the “Cys-loop” superfamily of ligand-gated ion channels that includes nicotinic ACh receptors (see below), glycine, and serotonin (5-HT_3_) receptors. GABA type A (GABAA) receptors are the major inhibitory neurotransmitter receptors in mammalian brain. These receptors are pentameric proteins composed of different subunits surrounding a central chloride ion-selective channel gated by GABA. Although many different GABAA receptor isoforms might exist, the major adult brain isoform consists of *α*1, *β*2, and *γ*2 subunits [180]. GABAA receptors are involved in various kinds of behavioral regulation, including sexual behavior [181], anxiety [182, 183], fear extinction learning [184], and object recognition memory [185]. These receptors also represent important pharmacological targets for treatment of several disorders, including epilepsy, anxiety, and alcoholism [186].

The GABAA receptor *γ*2 subunit is palmitoylated on unique cysteine residues localized within a major intracellular domain [187]. Functional analysis showed that suppression of *γ*2 subunit palmitoylation is critically involved in the synaptic clustering as well as in the regulation of the expression levels of GABAA receptors at the cell surface of cultured hippocampal neurons. Palmitoylation can also regulate trafficking and postsynaptic accumulation of GABAA receptors [171, 187]. The Golgi-resided palmitoyl acyltransferase DHHC3 (GODZ) was identified as a PAT responsible for the GABAA receptor *γ*2 subunit palmitoylation in a heterologous system and in neurons [171, 187, 188]. Combined results of these studies also demonstrated that GODZ plays an important role in an assembly and functional regulation of GABAergic inhibitory synapses. In the later study, the significant role of GODZ in the palmitoylation of GABA receptors was confirmed by creating DHHC3 knock-out mice [189].

### 4.3. Nicotinic Acetylcholine Receptors

As GABAA receptors, nicotinic ACh receptors (nAChRs) belong to the “Cys-loop” superfamily of LICs. Mammalian nAChRs contain five subunits divided into the alpha (*α*2–*α*7, *α*9, and *α*10) and beta (*β*2–*β*4) subfamilies, which assemble into both heteromeric and homomeric pentamers [190]. Thus there are a lot of subunit combinations leading to many different nAChR subtypes with various expression patterns, diverse functional properties, and differing pharmacological characteristics [191]. The nAChRs mediate excitatory neurotransmission at the neuromuscular junction, and at defined synapses in the brain, where they are often involved in the modulation of neurotransmitter release [192]. The cell surface expression of nAChRs is known to be modified during nicotine dependence and multiple disorders of the nervous system [193, 194].

Neuronal *α*4, *β*2, and *α*7 nAChR subunits have been found to be palmitoylated [195, 196]. Suppression of *α*4/*β*2 and homomeric *α*7 nAChR palmitoylation with bromopalmitate leads to the drastic reduction of the ligand binding, suggesting that palmitoylation might be directly involved in the formation of the ligand binding site during nAChR assembly in the ER [195]. Cysteine 273 in the cytoplasmic loop between transmembrane domains 1 and 2 (M1–M2) of the *α*4 nAChR was identified as a putative palmitoylation site [197]. Replacement of this cysteine by serine resulted in a nonpalmitoylated nAChR mutant and led to increased surface expression of receptor accompanied by decrease in the total expression. It is noteworthy that the functional activity of nonpalmitoylated *α*4 nAChR was not affected. Thus, palmitoylation of the *α*4 nAChR may be critically involved in the regulation of total and cell surface receptor expression.

### 4.4. P2X7 Adenosine Receptors

The P2X7 receptor is a trimeric ion channel gated by the extracellular ATP. This receptor is expressed in different tissues, including CNS. The P2X7 receptor received particular attention as a potential drug target because of its possible involvement in multiple neurological disorders, such as stroke, epilepsy, neuropathic pain, multiple sclerosis, and Alzheimer's disease [198]. In addition, recent studies demonstrated that P2X7Rs can be involved in the regulation of the pathophysiology of psychiatric disorders, including mood disorders and depression [199].

It has been shown that P2X7R is palmitoylated and that palmitoylation is responsible for association of receptor with lipid rafts at the plasma membrane [200]. The P2X7R contains 16 conserved cysteines within its intracellular domain. While cysteine residues Cys4, Cys5, Cys363, and Cys388 are not modified by palmitoylation, two separate regions of the C-terminal domain are important for P2X7R palmitoylation. The juxtamembrane cysteines in positions 371, 373, and 374 together with Cys477, 479, 482, 498, 499, 506, 572, and 573 were all implicated in palmitoylation. Functionally, palmitoylation is required for P2X7R maturation and exit from the ER, indicating that palmitoylation may play a role in the macromolecular organization of this receptor [200].

## 5. Conclusion

Data on the functional roles of palmitoylation summarized in the current review demonstrate that palmitoylation could be critically involved in the regulation of the neurotransmitter receptor's functions (Table 1). For GPCRs, these include binding of agonists/antagonists, targeting to the lipid microdomains, G protein coupling, trafficking, desensitization/internalization, sequestration, and phosphorylation (Table 1). One central function of palmitoylation of LICs is regulation of receptor trafficking. In addition, palmitoylation of ionotropic receptors can modulate a ligand binding, phosphorylation, internalization, protein/protein interaction, and synaptic clustering (Table 1). All these processes are critically implicated in the regulation of the GPCRs and LICs functional activities, although the specific role of palmitoylation differs depending on the particular receptor. Multiple findings also show that receptor palmitoylation plays differing functional roles at different receptors, suggesting that there is no common function applicable to all neurotransmitter receptors. Therefore, an analysis of the functions of palmitoylation is necessary for each individual receptor in order to understand its signaling mechanism.

Taken together, palmitoylation of neurotransmitter receptors is critically implicated in a control of a variety of important cellular processes, such as signal transduction and synaptic clustering [10, 11, 171, 187]. More importantly, pathological alterations in palmitoylation are often accompanied by severe neuronal disorders, such as Alzheimer's and Huntington's disease, schizophrenia, and mental retardation [18, 201]. This implies the importance of the receptor palmitoylation as a potential therapeutic target for the treatment of multiple neuronal diseases.

## Figures and Tables

**Table 1:** Palmitoylation sites and functions of neurotransmitter receptor palmitoylation.

Receptor type	Receptor subtype	Palmitoylation site	Palmitoylation function	Responsible DHHC	Reference
*G protein-coupled receptors*					
Adrenergic	*α*1AR	C120	Receptor-mediated signaling and receptor expression	DHHC21	[58, 59]
	α2AR	Cys442	Receptor downregulation followed by chronic agonist exposure	Unknown	[54, 57]
	*β*2AR	Cys341	Receptor phosphorylation and *β*-arrestin binding; coupling to the AC-mediated signaling and desensitization		[8, 47, 49–51]
		Cys265	Receptor trafficking and localization	DHHC9, DHHC 14, DHHC 18	

Serotonin	5-HT_1A_	Cys417, Cys420	Gi protein coupling/effector signaling, G*βγ*-mediated signaling	Unknown	[66]
	5-HT_4_	Cys328, Cys329	Regulation of constitutive receptor activity	Unknown	[68]
	5-HT_7_	Cys404, Cys438, Cys441	Regulation of constitutive, Gs-mediated receptor activity	Unknown	[68, 75]

Dopamine	D1R	Cys347, Cys351	Receptor internalization and functional activity	Unknown	[93, 94]
	D2R	C443	Receptor surface expression and stability	DHHC3, DHHC8, DHHC4	[95–97]
	D3R	C400	Receptor localization on the plasma membrane, endocytosis, agonist binding	Unknown	[98]
	D4R	Cys467	Receptor cell surface expression, signaling and endocytosis	Unknown	[100]

Vasopressin	V1a	Cys371, Cys372	Receptor conformation and phosphorylation	Unknown	[108]
	V2R	Cys341, Cys342	Receptor trafficking and localization on the plasma membrane, agonist-dependent receptor sequestration/internalization, and endocytosis	Unknown	[109–111]

Adenosine	A(1)AR	Cys309	Receptor-effector coupling, agonist-induced internalization/downregulation	Unknown	[118]
	A2BAR	Cys311	Receptor conformation and interaction with different proteins related to sorting and signaling	Unknown	[121]
	A(3)AR	Cys305	Receptor recycling to the plasma membrane after agonist removal	Unknown	[119]

Opioid	*μ*-Opioid	C170	Receptor homodimerization and G protein coupling/activation	DHHC3, DHHC4	[126–128]
	*δ*-Opioid	Unknown	Receptor trafficking and/or localization	Unknown	[129]

Cannabinoid	CB(1)	Cys415	Receptor conformation, trafficking and localization	Unknown	[6, 136]

Acetylcholine	Muscarinic	Cys457	Receptor interaction with G protein	Unknown	[143]

Neuropeptide Y	Y(1)	Cys337	Receptor conformation, G protein coupling, desensitization	Unknown	[149]

Melatonin	MT(1), MT(2)	Cys7.72, Cys7.77	Receptor functioning toward cAMP inhibition	Unknown	[156]

*Ligand-gated ion channels*					
Glutamate	NMDA	Cysteine clusters in the C-terminus of GluN2A and GluN2B subunits. The first cluster—GluN2A: C848, C853, and C870; and GluN2B: C849, C854, and C871. The second cluster—GluN2A: C1214, C1217, C1236, and C1239; and GluN2B: C1215, C1218, C1239, C1242, and C1245	Receptor retention in the Golgi apparatus, trafficking and surface expression/internalization	DHHC3	[4, 176, 177]
	AMPA	C610, C836	Subunit-specific receptors regulation and trafficking	DHHC2, DHHC3	[170, 172, 202]
	Kainate	C827 and C840	Receptor insertion and stabilization at the cell surface	Unknown	[178, 179]

GABA	GABAA	Multiple cysteine residues within the major intracellular domain of gamma2 subunit	Synaptic clustering of receptor, cell surface expression, and trafficking	DHHC3, DHHC7	[187–189]

Acetylcholine	Nicotinic *α*4	Cys273	Receptor total and cell surface expression	Unknown	[197]

Adenosine	P2X7	Cys371, 373, 374, 477, 479, 482, 498, 499, 506, 572, 573	Receptor trafficking and plasma membrane localization; receptor macromolecular organization	Unknown	[200]
